# Dreams as a source of supernatural agent concepts

**DOI:** 10.3389/fpsyg.2015.00283

**Published:** 2015-03-19

**Authors:** Patrick McNamara, Kelly Bulkeley

**Affiliations:** ^1^Department of Neurology, Boston University School of Medicine – Veterans Affairs Boston Healthcare SystemBoston, MA, USA; ^2^Graduate Theological Union in Berkeley, BerkeleyCA, USA

**Keywords:** dreams, creativity, supernatural agents, religion, REM sleep

## Abstract

We present a theory of the creativity of dreams as well as psychopathology of religious delusions with respect to production of fundamental forms of religious cognition—specifically supernatural agent (SA) cognitions. We suggest that dream cognitions are particularly efficient at producing highly memorable and impactful experiences with SAs because dreams involve three processes that are prerequisites for the generation of god concepts: (1) mental simulations of alternative realities, (2) theory of mind attributions to the extra-natural dream characters and divine beings, and (3) attribution of ultimate value (exemplified by ‘good spirit beings’), and dis-value (exemplified by demonic monsters) to the supernatural dream characters. Because prefrontal cortex is deactivated during rapid eye movements (REM) sleep agentic impulses and internally generated ideas are not reliably attributed to Self or dreamer. Instead an exaggerated degree of agency is attributed to these supernatural dream characters who are then embedded in stories in dreams and in myths of waking life which explain their supernatural abilities. These dream-based SAs are salient characters that are processed in sleep-related memory systems according to rules of Lleweelyn’s ancient art of memory model and therefore more easily remembered and reflected upon during waking life. When REM sleep intrudes into waking consciousness, as is the case with some forms of schizophrenia, religious delusions are more likely to emerge.

## Introduction

In this paper we highlight dreams as a source of creativity-specifically in the realm of religious ideas. We also detail a role for dreams in production of religious delusions associated with certain forms of psychopathology such as schizophrenia. For over a century anthropologists working in every part of the world have amply documented the importance of the dream as a primary source for religious ideas and practices of traditional peoples (Tylor, 1870; Lincoln, 1935; Grunebaum and Caillois, 1966; Tedlock, 1992). In many traditional societies dreams were considered direct, experiential evidence of a spirit realm (see reviews in Bulkeley, 2008a; Mittermaier, 2010). Many trial peoples interpreted their dreams as cases where the dreamer’s soul wanders outside the body and communes with the gods and spirit beings of the spirit world (e.g., Gregor, 1981; Lohmann, 2003a,b,c). Harner (1984), reports that the South American Jívaro consider dreams to be populated by spirit beings who often are, or become the focus of their sacred rituals and stories. The Zuni and Quiché Maya traditions studied by Tedlock (1992) see dreams as direct communications from sacred ancestors. The Tikopia (Firth, 2001) in Polynesia see many dreams as direct communication of spirit beings to the dreamer.

The characters populating these dreams can be described, in the language of the cognitive science of religion, as *supernatural agents* (SAs): non-human entities with autonomous powers and intentions. Their bodies may not be clearly seen, but they do have minds and they frequently have unusual powers far beyond the capabilities of ordinary agents (e.g., flying, time-travel, mind-reading; Boyer, 2002). More specifically, these SAs can be characterized as “minimally counterintuitive.” “Counterintuitive (CI)” means their unusual abilities violate our intuitive assumptions about what normal agents can and cannot do. They are “minimally” CI because they violate *some* of our intuitive assumptions, but not *all* of them. They are just CI enough to draw attention and arouse curiosity without overwhelming comprehension, making them easier to remember, and describe to other people. CI concepts can be scored in narratives (Barrett, 2008). If we use Barrett’s scoring criteria for CI concepts we find that dreams are filled with them (McNamara, unpublished data).

Boyer argued that the CI properties of SAs makes them more memorable. If an agent is too CI and violates our assumptions in too many ways, we will have more difficulty recalling its characteristics and telling other people about it (a crucial factor in how religions spread over time). However, if an agent is not CI enough, it will not make a strong enough impression on awareness, and it will thus sink into general forgetfulness. The notion of “minimally counterintuitive” SAs is intended to designate a cognitively optimal set of features that give god concepts a strong, innately compelling appeal to the human mind.

Despite the abundance of anthropological evidence linking people’s beliefs in SAs with dream experiences, few efforts have been made to explain the brain/mind processes connecting dream cognitions and religious cognitions. In this paper we present evidence in favor of the hypothesis that *supernatural agent cognitions are significantly influenced by nightly dream content patterns*. We present several interrelated strands of evidence to support the plausibility of this idea and explain the mechanisms involved. We begin with an overview of the neurobiology of rapid eye movements (REM) and non-rapid eye movement (NREM) sleep states insofar as this neurobiology relates to dream cognitions. We then review historical and cross-cultural evidence linking dream cognitions to SA cognitions. We conclude with a presentation of a theory as to how dream cognitions generate SA cognitions.

## REM and NREM Sleep States

When measured electrophysiologically, sleep (in humans) is composed of four major stages. The first three (N1–N3) stages constitute NREM sleep, and are best described by polysomnographic and electroencephalographic (EEG) criteria. The fourth stage is not part of NREM sleep and is characterized by periodic or phasic bursts of REM under the closed eyelids. REM sleep is indicated by EEG data, electroculographic activity, and a reduction in electromyographic (EMG) activity. EMG electrodes are usually placed under the chin, and indicate when the muscle paralysis characteristic of REM sleep occurs (Rechtschaffen and Kales, 1968).

Rapid eye movements and NREM sleep states alternate throughout the night in a period of about 90 min. Stage N1 is a transitional stage to N2 or light sleep. N3 is composed of slow-wave sleep which is particularly potent during the childhood phase of development. NREM stages dominate the first third of the night, while REM dominates the last third (Carskadon and Rechtschaffen, 2000). A sleep cycle is defined as a period of NREM sleep followed by a period of REM sleep. During a single night, a person will progress through three or four NREM–REM sleep cycles, each lasting 90–110 min and becoming progressively longer through the night. Usually, the NREM period will last about 80 min or longer, followed by a REM period that can be as short as 1 min in the early cycles. Toward the morning REM periods can last as long as 45 min.

Rapid eye movement sleep accounts for about 22% of total sleep time in humans. The cortex, particularly the limbic region, is very highly activated in REM. The phasic aspects of REM, such as intermittent muscle twitching, autonomic nervous system (ANS) discharges, and REM, occur in some mammals in association with bursts of pontine-geniculo-occipital waves. Mammals (with the possible exception of humans) also exhibit a theta rhythm in the hippocampal formation during REM night.

Rapid eye movement’s tonic characteristics are a desynchronized EEG, penile erections, and atonia of the antigravity muscles. Its phasic characteristics include bursts of REM under the closed eyelids, myoclonic twitches of the facial, and limb muscle groups, increased variability in heart rate, respiration, and blood pressure, and ANS discharges. Other correlates of REM include effects on release of selected hormones – especially growth factors. Virtually all mammals (with some exceptions such as certain sea mammals) exhibit both SWS and REM sleep.

Rapid eye movements sleep demonstrates high activation levels in limbic/amygdaloid sites, in dopaminergic, and cholinergic circuits but deactivation of dorsolateral prefrontal cortex sites (Maquet et al., 1996; Braun et al., 1997; Maquet and Franck, 1997; Nofzinger et al., 1997; Hobson et al., 1998; Dang-Vu et al., 2007; Hobson, 2007), as well as cessation of activation in the noradrenergic locus ceruleus and the serotoninergic raphe nucleus. Stage N2 sleep involves higher activation levels in filiative paralimbic regions in the cortex relative to REM sleep and to the deep sleep of NREM stage ‘slow wave sleep.’ Larson-Prior et al. (2009) examined MRI-blood oxygen dependent signal functional connectivity using conventional seed-based analyses in several cortical sites as healthy young adults entered NREM sleep from wakefulness. Functional connectivity was maintained in cortical networks in NREM sleep. Thus both NREM N2 stage sleep and REM sleep are characterized by brain activity patterns that can support complex cognitive processes.

Dream content differs to some extent across REM and NREM sleep states, and so SA cognitions may also differ in relation to sleep states. For example, using the standardized / Hall Van de Castle dream scoring scales (Hall and Van de Castle, 1966; Domhoff, 1996), dreams obtained from N2 stage sleep tend to be more thought-like, less bizarre, and less emotional than dreams obtained from REM (Domhoff, 1996). McNamara et al. (2005) scored REM and NREM dreams and wake reports for number and variety of social interactions. We found that aggression levels were higher in REM vs. NREM, or wake reports and that conversely, dreamer-initiated friendliness was more characteristic of NREM than REM. These findings were recently replicated using standard polysomnographic and EEG sleep-staging techniques (McNamara et al., 2010). One straightforward hypothesis flowing from these data with respect to SA cognitions is that friendly spirits would more often though not exclusively be encountered in NREM dreams and less friendly or demonic spirits would be encountered more often, though not exclusively in REM dreams.

Rapid eye movements physiology can intrude into daytime consciousness and when it does it may create delusional states. It is well established that sleep deprivation reliably produces compensatory attempts to enter REM throughout the day resulting in REM microsleeps as well as reports of dreamy states and dissociative symptoms.(Limosani et al., 2011; Palagini and Rosenlicht, 2011) The role of REM-irruptions into waking consciousness is well-recognized in narcolepsy where the associated experience is the production of hypnagogic hallucinations. Mahowald et al. (2011) and van der Kloet et al. (2012) review a range of evidence which demonstrates that dissociative symptoms such as absorption, derealization, depersonalization, and other symptoms associated with a range of psychiatric disturbances originate from sleep. REM intrusion has been reported to contribute to symptoms and delusional states in schizophrenia (Gottesmann and Gottesman, 2007; Cohrs, 2008). Religious delusions and unusual religious experiences are more frequent among schizophrenic patients with positive symptoms compared to that of the general population (Siddle et al., 2002; Kimhy et al., 2005; Green et al., 2006; Huguelet et al., 2006; Mohr et al., 2010). Religious delusions are erroneous beliefs that a SA is shaping the experiences of the rooted patient. The content of religious delusions are typically persecutory (e.g., ill-intending evil spirits) and involve alterations in sense of self including grandiosity or extreme self-denigration and these delusions are even more resistant to rational disconfirmation than other types of delusions.

Having reviewed the fundamental neurology of REM and NREM sleep states as well as ways in which REM and dreams may impact psychopathology we turn now to evidence for the view that SA cognitions are causally related to dream cognitions.

## Dreams in History of Religions

According to early anthropologists like Tylor (1870) it seems probable that humans first entertained the idea of a spirit realm because they directly experienced such a realm in their dreams. Researchers working in many different parts of the world have found that people in traditional societies treat dreams as the sources of their religious ideas, including their concepts of their gods and other supernatural beings (e.g., Gregor, 1981 on Amazonia; and Lohmann, 2003a,b,c on Oceania; and Tonkinson, 1974, on Aboriginal Australia; Shweder and LeVine, 1975; Keesing, 1982; Shweder and Bourne, 1982; Shaw, 1992; Hollan, 2003; Robbins, 2003). It is likely that ancestral populations also treated them as such. Dreams were considered proof of the gods and a spirit realm since dreams were involuntary and emotionally vivid experiences that involved the dreamer’s soul encountering other beings including long deceased relatives and so on. Dreams have therefore played a major role in the historical evolution of religions.

Although the first glimmers of religious consciousness likely occurred in tandem with Neanderthal burials some 100,000 years before the present, the first clear signs of the emergence of SAs occurs in the hundreds of cave art sites that have been dated to the Upper Paleolithic. In the Chauvet cave that dates to some 28,000 years ago, there are many images of therianthropes or human-animal hybrids that very likely depicted SAs. Ancient rock art from all over the world also very often depicts masked human forms and therianthropes. The Tassili rock art of the Sahara depict therianthrope SAs that date to a time (the Neolithic and perhaps earlier) when the Sahara still contained grasslands where flocks of animals could be hunted. The rock art of the San of South Africa depict larger than life hunters garbed in animal masks and skins. The rock art of native tribes of Australia that date back many 1000s of years also depict similar therianthropes.

The examples could be multiplied many times over. This suggests that SA figures first occurred in the visionary dreams of the people who painted these images. A number of scholars have argued that traditional diviners/healers known as *shamans* may have painted some of these images (Lewis-Williams, 2002; Hayden, 2003). Current ethonographic evidence (Eliade, 1964) suggests that shamans contact their spirit helpers and divine being via two methods: ecstatic states induced by entheogens, or other methods and via dream states. We have evidence that that is how the shamans of the upper paleolithic derived their god figures as well. Right at the beginning of the upper Paleolithic we have an image of a shaman in headdress with antlers and animal skins at Fumane cave in Italy dated to 35,000 years ago. The “sorcerer” image from the cave at Les Trois Freres shows a man wearing animal skins, arms outstretched like a horse rearing with a reindeer antler headdress, owlish eyes, bear paws, and human beard. Another image can only be seen by lowering oneself down via rope into the famous ‘shaft’ at Lascaux. The image should really be termed a composition as it depicts some sort of interaction between a wounded bison and a therianthropic figure lying below the bison. The figure is of a man with an erection and a bird-like head. Standing upright next to the man is his staff. The staff has the same sort of bird perched on top. The bird is probably the totem animal for this shaman. Many shamans believe they become birds when they fly into the heavens when traveling to the spirit world. The fact that the man has an erection suggests that he is dreaming in REM sleep (dreams and erections invariably occur in association with REM). The shaman is dreaming the sacrifice of the bison and the bison is considered a sacred spirit being.

We have seen that dreams likely played a role in creation of religious consciousness at the dawn of the human adventure. They have continued to play a role in religious consciousness ever since (Bulkeley, 2008a,b). All the world’s religious traditions have pointed to dreams as spiritual events of extreme importance in the transformation of the self and with respect to communication with the spirit world (see O’Flaherty, 1986; Jedrej and Shaw, 1992; Irwin, 1994; Young, 1999; Mittermaier, 2010). This is not the place to document the full extent of the multiple roles of dreaming in the world’s religions, but two highlights can be mentioned to suggest the close connection between dreaming experience and religious concepts.

First is a Biblical story, sacred to Jews, Christians, and Muslims alike, about the patriarch Jacob, and his dream theophany, reported in Genesis 28. The dream occurred after Jacob had tricked his blind father Isaac into giving him the blessing that should have gone to his older brother, Esau. Enraged at this trickery, Esau made a vow to kill Jacob. However, their mother, Rebekah, overheard Esau, and she arranged for Jacob to visit relatives in a distant village, giving him the opportunity to escape before his brother can find and murder him. Away Jacob went, out into the wilderness. One night during his solitary journey through the deserts of ancient Canaan he lay down, placed a stone under his head for a pillow, and went to sleep:

“And he dreamed that there was a ladder set up on the earth, and the top of it reached to heaven; and behold, the angels of God were ascending and descending on it! And behold, the Lord stood above it, and said, ‘I am the Lord, the God of Abraham your father and the God of Isaac. The land on which you lie I will give to you and to your descendants; and your descendants shall be like the dust of the earth, and you shall spread abroad to the west and to the east, and to the north, and to the south; and by you and your descendants shall all the families of the earth bless themselves. Behold, I am with you and will keep you wherever you go, and will bring you back to this land; for I will not leave you until I have done that of which I have spoken to you.”

(Gen. 28:12–15, RSV; Metzger and Murphy, 1991).

Jacob awoke from this dream filled with wonder, surprise, and fear. “Surely the Lord is in this place; and I did not know it,” he says to himself. “How awesome is this place! This is none other than the house of God, and this is the gate of heaven.” At a time of deep personal uncertainty, with his life in danger and his future totally unknown, Jacob experienced an overwhelming dream vision of heavenly angels, with the reassuring words of God to soothe his fears and rouse his procreative energies.

Whether or not there was really a man named Jacob who actually had this dream, we can recognize in this Biblical story an early awareness of the wonder-working power of dreaming and its influence on religious beliefs about SAs.

A second example, not nearly as well known, comes from an ancient Japanese Buddhist context. The 11th century C. E. woman known as Lady Sarashina (her real name is not known) recorded a number of dreams in her diary, translated as *As I Crossed a Bridge of Dreams* (Morris, 1971). Toward the end of her life, after her husband had died and her hopes for working in the romantic world of the Imperial palace had been dashed, she described the following, which occurred on “the thirteenth night of the Tenth Month of the third year of Tenki”:

“[I] dreamt that Amida Buddha was standing in the far end of our garden. I could not see him clearly, for a layer of mist seemed to separate us, but when I peered through the mist I saw that he was about six foot tall and that the lotus pedestal on which he stood was about four feet off the ground. He glowed with a golden light, and one of his hands was stretched out, while the other formed a magical sign. He was invisible to everyone but me. I had been greatly impressed but at the same time frightened and did not dare move near my blinds to get a clearer view of him. He had said, ‘I shall leave now, but later I shall return to fetch you.’ And it was only I who could hear his voice.”

(Morris, 1971, 107).

This dream of intimate communion with a SA made a big impact on Lady Sarafina—“thereafter it was on this dream alone that I set my hopes for salvation.” Like Jacob, her waking life seemed bleak and hopeless, and in that emotional context she has a vivid, highly memorable dream of a beneficent deity who provides reassurance and a revived sense of hopefulness about the future.

Many more cross-cultural examples could be offered, but these should be sufficient to indicate the strong and widely felt impact that dreams have had on people’s religious and spiritual concepts throughout history.

## Dreams as a Source for Religious Cognitions

We have summarized the view that dreams have been a key source for religious cognitions and experiences throughout the history of religions. We now present a theory as to how dreams act as a source for religious cognitions.

First, for many people dreams present direct evidence of a spirit realm and of disembodied spirits. For example, in so called visitation dreams the dreamer sees a beloved friend or relative who in waking life has died. In the dream the loved one delivers a message to the dreamer that he or she is well. The dreamer awakens with an absolute conviction that the person in the dream was really, truly the loved one. The “visitation” is often described as so real and lifelike that grief over the death of the loved one is significantly diminished and the dreamer feels that contact with the deceased is real.

Second, many people experience direct contact with a supernatural being in their dreams. The dream of Lady Sarafina mentioned above is a good example of such a dream. The contact with a divine figure is once again experienced as intensely real, awe inspiring and definitive. The impact of this contact with a divine being is so significant for the dreamer that it changes his or her life for months or years thereafter.

We suggest that dream cognitions are particularly efficient at producing highly memorable and impactful experiences with SAs because dreams involve three processes that are prerequisites for the generation of god concepts: (1) mental simulations of alternative realities, (2) theory of mind (ToM) attributions, and (3) computation of ultimate value (exemplified by ‘good spirit beings’), and dis-value (exemplified by demonic monsters).

### Dreams as Mental Simulations

Many theorists have attempted to characterize the species of cognition one sees in dreams. One proposal that has received some preliminary empirical support is that many dreams specialize in mental simulations of worlds or states of affairs different from the waking world. Of course the spiritual realm is one such “world” for most religious traditions. McNamara et al. (2001) has proposed that mental simulations of alternative worlds or of ultimate realities can be either oriented to the past, in which case they are counterfactuals, or they can be oriented toward the future, in which case they are examples of prospection. A third form of simulation important for dreams is projection of the self as in ToM attributions. We will discuss ToM attributions below. Here we remark on past- and future-oriented mental simulations that occur in dreams.

Dreams very likely play a role in “episodic prospection” or “episodic future thought” (Spuznar, 2010) such as planning, development of goals, generation of desires, mental simulations of possible worlds, imagination of future scenarios, and contingencies, and daydreaming about possibilities. For example, in dreams the dreamer is virtually always desiring, planning, imaging, plotting, striving, simulating possible worlds, and in general aiming at some future outcome. Dreams are goal driven and that is why they are sometimes spoken of as narratives, stories, or episodes.

McNamara et al. (2001, 2002) has discussed past-oriented counterfactual simulations in dreams. Counterfactuals in logic characterize conditions under which some state of affairs might have been true. In philosophy and in language semantics counterfactuals have been used to characterize modal event logics and to use possible world scenarios to evaluate formal languages. In economics counterfactuals are crucial to a range of theories concerning risk and decision-making rules under conditions of regret and attempts to avoid losses. In psychology and cognitive neuroscience counterfactuals are treated as mental simulations of possible worlds or states of affairs.

Regardless of the various ways in which counterfactuals are treated in these disparate intellectual disciplines, the common denominator is that they are mental simulations of what might have happened if I had chosen differently or performed slightly differently. Their appearance in dreams have been documented by McNamara et al. (2002).

### Theory of Mind Attributions

Theory of mind attributions are ubiquitous in dreams. Schweickert and Xi (2010) found an average of nine instances of ToMs per dream in a set of dreams they studied. Thus dreams satisfy one of the main requirements that many theories of religious cognition demand for thinking about SAs, namely the capacity to generate ToM attributions. But we believe that the generation of SAs requires more than mental simulation of possible worlds and/or the capacity to do ToM attributions. To ascribe religious significance to a super-natural being in a dream or wake state I need to do more than to simply cognize or realize that that being has a mind like mind, I also have to reverence or fear (or both) that being; I have to in short impute value or significance to that being.

### Computation of Value

Although many authors have asserted that the proximate origins of god concepts are ToM attributions, it is not clear to us that the mind-reading system, by itself, produces the kinds of SAs that generally populate religious ideologies or traditions. It is not clear how hierarchical meta-representations or processes like these yield a deity that we feel awe and reverence for. For most human beings throughout history reading the god’s minds was not a primary goal. Rather it was establishing a relationship with the god that mattered because that god represented power and one’s highest values. For most human beings gods demand worship or reverence and often fear. A human being cannot be in the presence of a thing of immense value and power without some sort of emotional response like reverence, devotion, or commitment.

It is this latter stance of *commitment to the god* that is missing from ToM and related accounts of religious cognition. We not only attempt to know and be known by a god. We attempt to relate to them, to unite ourselves to them, or in the case of demonic SAs, we attempt to flee/avoid them. There is an emotional response because matters of ultimate concern are involved where religious cognition is concerned.

We have mentioned above how dreams typically involve the dreamer pursuing or trying to attain to some desired state or to avoid a fearful outcome. These are obviously instances of perception of value and pursuit of value. The neurobiology of REM and NREM sleep states is now understood to involve forebrain mesocortical dopaminergic systems that directly compute value and dis-value (Perogamvros and Schwartz, 2012). In addition, with respect to REM itself, replicated findings have shown decreased anterior-posterior EEG coherence at gamma frequencies during REM vs. NREM sleep (Perez-Garci et al., 2001; Corsi-Cabrera et al., 2003, 2008) as well as in phasic vs. tonic REM sleep (Corsi-Cabrera et al., 2008). Coherence decreases appear to reflect limbic and amygdalar activation during REM sleep that is particularly enhanced during phasic REM sleep. Limbic and amgdalar systems are core systems involved in the computation of value. REM sleep demonstrates high activation levels in midline, ventral paralimbic areas, including medial prefrontal cortex, along with deactivation of lateral prefrontal cortices (Maquet et al., 1996; Braun et al., 1997; Maquet and Franck, 1997; Nofzinger et al., 1997, 2004) identify a midline anterior paralimbic areas that includes both subcortical areas such as basal forebrain, hypothalamus, ventral striatum, and hippocampus as well as paralimbic cortices such as insula, anterior cingulate, orbitofrontal cortex, and supplementary motor area. Reactivation of these paralimbic area, as well as deactivation of dorsal prefrontal regions with the transition into REM sleep has now been widely replicated (reviewed in Maquet et al., 2005; Nofzinger et al., 2006; Pace-Schott et al., 2009).

In short, the phenomenology of dreaming and the neurobiology of sleep suggest that dreaming cognition reflects the processing of value-related information. The fact that both dreams and religious cognitions are particularly concerned with the computation of ultimate values provides one more empirical link between them.

To summarize, dreams exhibit the three core functional capacities that are required for production of SAs: mental simulations of other worlds, ToM attributions, and computations of ultimate value.

This means all humans are endowed with brains innately primed to daily generate god concepts in dreaming.

#### Mechanisms

But how do dreams do it? At a minimum, we assume that the dreaming mind/brain constructs SAs via the ascription of agency to selected “other” characters in dreams. When a superabundance of agential attributes are attributed to these characters we get a divine being. Now the reason the dreamer ascribes agency to other characters in dreams is due to change in the normal way in which agency is constructed in the normal course of daily events in waking life. We discuss the construction of the sense of agency in waking life next.

#### Cognitive Models of Agency

Most of us can think inward thoughts and know that we are the source of those thoughts. However, schizophrenics, people with active bipolar disease and other individuals with neuropsychiatric disorder do not understand or believe that they are the source of the thoughts in their heads. How does this happen?

To see how someone can fail to see their own thoughts or actions as issuing from themselves we need to first say a little about how the predictive brain works to confirm its own actions. Most neuroscientists believe that the way in which this is accomplished is that whenever we think a thought or issue an action the brain issues an unconscious prediction of the predicted effects of that action or thought in the real world. If those predicted effects occur then the brain gets the confirmation it needs to conclude that the action or thought came from itself. If unpredicted effects occur then the brain needs to do further work to figure out whats going wrong. In a neuropsychiatrically impaired individual that further work is impeded or not done at all so the brain concludes that the thought or action must have come from outside the self or from some other being.

In short, the subjective experience of ownership of one’s own thoughts and of control over sensory events emerges from an unconscious comparison between intentional and predicted /anticipated effects of one’s actions: if there is a match, there is an increased tendency to experience the effect as self-caused, whereas a mismatch between anticipated and actual effect increases the tendency to attribute the effect to an external cause. The brain uses sensory feedback mechanisms wherein sensory effects of the action are assessed, to determine if there is a match or mis-match between intended vs. predicted effects of the action. If predicted effects occur then sensory feedback will be attenuated relative to a case where anticipated effects do not occur.

In REM dreaming we know that the prefrontal cortex activation levels are downregulated relative to waking levels. It is entirely possible therefore that the normal attenuation process of sensory feedback does not occur as efficiently as it does in waking. Thus the dreamer may conclude that the source of the efferent signals that arise from intentional scenarios that occur in dreams is not himself, so he therefore ascribes it to some other animate being in the dream. If there is no match between predicted and actual effects (and there cannot be in the dream) then there is little or no basis for ascribing intentional actions to the self and a corresponding increased pressure to ascribe them to another character who is invested with a very strong sense of agency-thus a divine being.

Although we know of no direct empirical studies on dream content that unequivocally links the REM-associated diminution in agency in the dreamer with a corresponding increase in agency in a special OTHER dream character, we think examples abound in the dreams we all have each night. Consider the ubiquitous dream theme of the dreamer attempting to escape from a pursuer. The dreamer seems unable to run fast enough to evade the pursuer or find ways to stop the pursuer who becomes more frightening as he advances on the dreamer. Or consider the norms on dream content variables derived from the Hall and Van de Castle standardized scoring system used now in hundreds of dream content studies (see Domhoff, 2003 for review). “Victimization” of the dreamer is reported in greater than 65% of dreams; self negativity appears in 66% of dreams; dreamer-involved success is found in less than 50% of dreams and misfortunes for the dreamer occur in greater than one third of dreams. Clearly most dreams involve setbacks or a diminution of agency in the dreamer. New research should assess potential links or quantitative correlations between the appearance of misfortune for the dreamer and greater success, power, or vividness for an opposing dream character.

Once a dream character has been invested with magical powers of agency and power in a dream these privileged characters then need to be processed specially so that they are selectively remembered as “special” during waking life. Llewellyn’s recent theory of the ways in which REM and NREM sleep states use classical mnemonic processes to encode memories can help explain why “special” beings are so often remembered as part of dreams: in Llewellyn’s model elaborative encoding in REM can, at least partially, be understood through ancient art of memory (AAOM) principles: visualization, bizarre association, organization, narration, embodiment, and location. Visualization of a divine being occurs in order to instantiate the required ascription of agency to a character after self ascription fails. Once visualization of a divine being occurs his or her special powers are put on display in a dream scenario thus forming bizarre associations with the figure. The encounter with the divine being, as in lady Serafina’s dream above, occurs in a special sacred location and thereby elicits intense emotion. Finally the encounter between the dreamer and the divine being is embedded in a narrative derived from the local culture that generally has the deity imparting some message to the dreamer.

## Conclusion

There can be little doubt that dream experiences have been thoroughly intertwined with the religious beliefs, practices, and experiences of people all over the world, throughout history. Although dreams are difficult to study scientifically, the sheer fact of their psychological and cultural ubiquity makes them an important topic for brain-mind research as well as for a scientific theory of religion. We have argued that new advances in the cognitive science of religion, particularly regarding SAs and the cognitive foundations of agency, can illuminate some of the neurological processes at work in dreaming that naturally lend themselves to attributions of special powers to “special characters/beings” in dreams and therefore to religious meaning and purpose.

We have also reviewed evidence suggesting that some psychiatric symptoms such as dissociative states and delusions have been linked with REM parasomnias and intrusion of REM into waking consciousness (due to sleep deprivation or to disease related breakdown in normal sleep architecture). In those psychiatric disorders that evidence sleep deprivation and REM fragmentation as well as religious delusions such as schizophrenia, the mechanism producing religious delusions may be illuminated by some of the links between dreams and SA cognitions we discuss in this paper.

It is beyond the scope of this paper to discuss possible methods of influencing, shaping, or guiding this religion-generating capacity of dreaming. We can point to examples like the dream incubation rituals performed at the ancient temples of the Greek healing god Askelpius, and suggest that these practices were effective because they skillfully channeled all mental and physical energies toward the healing process. The brain is not an isolated organ; every individual has been raised in a cultural environment that shapes the functioning of the mind in waking and in dreaming. Future research should consider exploring the cultural and psychological dynamics underlying these ritual practices.

## Conflict of Interest Statement

The authors declare that the research was conducted in the absence of any commercial or financial relationships that could be construed as a potential conflict of interest.
